# Expanding the genotypic spectrum of PCSK1 deficiency: A novel mutation in severe neonatal diarrhea

**DOI:** 10.1002/jpr3.70127

**Published:** 2025-12-12

**Authors:** Eleonora Saraceno, Angelo Corso Faini, Silvia Deaglio, Marianna Pellegrini, Raffaele Buganza, Francesca Giuliani, Pier Luigi Calvo, Michele Pinon

**Affiliations:** ^1^ Department of Public Health and Pediatrics Postgraduate School of Pediatrics, Regina Margherita Children Hospital, University of Turin Turin Italy; ^2^ Immunogenetic and Transplant Biology Unit Regina Margherita Children Hospital, AOU Città della Salute e della Scienza di Torino Turin Italy; ^3^ Clinical Nutrition and Dietetics Unit Regina Margherita Children Hospital, AOU Città della Salute e della Scienza di Torino Turin Italy; ^4^ Pediatric Endocrinology Unit Regina Margherita Children Hospital, AOU Città della Salute e della Scienza di Torino Turin Italy; ^5^ Neonatal Special Care Unit Regina Margherita Children Hospital, AOU Città della Salute e della Scienza di Torino Turin Italy; ^6^ Pediatric Gastroenterology Unit Regina Margherita Children Hospital, Azienda Ospedaliera‐Universitaria Città della Salute e della Scienza di Torino Turin Italy

**Keywords:** congenital diarrhea, endocrinopathy, genomic testing, PCSK1 mutation

## Abstract

Among congenital diarrhea and enteropathies (CODEs), proprotein convertase subtilisin/kexin type 1 (*PCSK1)* deficiency is a rare monogenic disorder, associated with severe neonatal diarrhea and polyendocrinopathies. We report an 18‐day‐old male neonate, born to consanguineous parents, presenting with persistent watery diarrhea, metabolic acidosis, and failure to thrive. Genetic analysis identified a novel homozygous c.709+1 G > A splicing mutation in *PCSK1*, predicted to disrupt protein function. Rapid genetic diagnosis enabled timely endocrine assessment and guided nutritional management, minimizing invasive procedures. Expanding the *PCSK1* mutational spectrum, this case highlights the pivotal role of next‐generation sequencing in neonates with refractory diarrhea and suggestive clinical history, facilitating early intervention and improved outcomes.

## 1 INTRODUCTION

Congenital diarrhea and enteropathies (CODEs) are a group of rare monogenic intestinal epithelial disorders characterized by early‐onset, severe, and persistent diarrhea with malabsorption, typically requiring intensive medical support in the neonatal period.^1^ Among these, proprotein convertase subtilisin/kexin type 1 (PCSK1) deficiency represents a particularly rare form impairing enteroendocrine cell (EEC) function. To date, approximately 30 cases have been reported, exhibiting a pleiotropic range of clinical manifestations including malabsorptive diarrhea, hypogonadotropic hypogonadism, altered thyroid and adrenal function, diabetes insipidus, and glucose metabolism abnormalities associated with elevated proinsulin levels.^2^

## 2 CASE REPORT

An 18‐day‐old male neonate was admitted to the emergency department due to persistent watery diarrhea and poor weight gain.

He was born 36 weeks and 5 days via cesarean section to consanguineous parents (second cousins), with a birth weight of 2700 g (74th percentile) and an Apgar score of 2 at 1 min and 9 at 5 min. Early hypoglycemia was managed with parenteral glucose supplementation. Despite exclusive breastfeeding postdischarge, subsequent poor weight gain prompted formula supplementation at 18th day of life. Shortly thereafter, he developed profuse watery diarrhea, worsening lethargy, and further weight loss.

Upon admission, the infant exhibited a body weight of 2480 g (4th percentile), signs of severe dehydration and metabolic acidosis (pH 7.08, HCO₃⁻ 8.7 mmol/L, BE −21.4 mmol/L), requiring urgent intravenous rehydration and acid–base correction. Given the severe clinical presentation, total parenteral nutrition (TPN) was initiated. Notably, stool output improved with fasting, suggesting osmotic/diet‐induced diarrhea.

Approximately 10 days later, enteral feeding with a 5% glucose solution was attempted to assess for carbohydrate malabsorption. In the absence of diarrheal stools following this attempt, an amino acid‐based formula was introduced on suspicion of cow's milk protein allergy. However, as enteral feeding was gradually increased to meet full nutritional requirements, watery diarrhea relapsed, requiring resumption of full TPN.

Extensive workup excluded infectious, anatomic, metabolic, renal, and immunologic causes. Consanguinity and early onset persistent diarrhea raised suspicion for monogenic enteropathy. Clinical exome sequencing (CES) focused on a panel of genes associated with CODEs and very early onset inflammatory bowel diseases (VEOIBDs) identified a homozygous c.709+1 G > A variant in the *PCSK1* gene. Familial segregation analysis, performed via Sanger sequencing, confirmed heterozygosity in both parents. In silico splicing prediction (SpliceAI^3^) indicated an 88% probability of donor‐site disruption, leading to aberrant splicing and potential loss of function. Following American College of Medical Genetics and Genomics (ACMG) criteria,^4^ the variant was classified as likely pathogenic (class 4).

Following the diagnosis of PCSK1 deficiency, comprehensive endocrinological, gastroenterological, and nutritional follow‐up was initiated due to the risk of associated endocrinopathies and early onset obesity. Endocrinological assessment revealed central adrenal insufficiency, requiring cortisone acetate therapy and micropenis, consistent with hypogonadism. The hypothalamic‐pituitary‐thyroid axis was preserved, and no clinical or biochemical evidence of diabetes insipidus was observed.

Along with the parenteral nutritional support, a gradual reintroduction of breast milk was performed, allowing partial weaning from TPN. At 3 months of life the patient was discharged with a body weight of 4620 g (5th percentile), a length of 55.4 cm (5th percentile), and a weight‐for‐length at 46th percentile, fed 40% by PN and 60% by breast milk, without diarrhea recurrence.

At two months postdischarge, catch‐up growth was satisfactory, with weight increasing to the 25th percentile and weight‐for‐length to 75th percentile. Enteral tolerance progressively improved, enabling complete TPN weaning by 6 months of age.

## 3 DISCUSSION

CODEs stand out within the heterogeneous landscape of “intractable diarrhea of infancy,” first described by Avery as chronic, unresponsive diarrhea requiring prolonged hospitalization. Advances in genetic testing, especially next‐generation sequencing, have improved outcomes through earlier definitive diagnoses and targeted management.^5^

In our case, early genetic testing, prompted by suggestive clinical features (consanguinity and intestinal failure with PN dependence), enabled the diagnosis of enteric dysendocrinosis due to PCSK1 loss‐of‐function.

PCSK1 deficiency belongs to the category of CODEs caused by EECs dysfunction. Other relevant genetic causes include NEUROG3, RFX6, ARX, and PERCC1, each characterized by distinct molecular mechanisms and systemic manifestations. A comparative overview is provided in Table 1 to support differential diagnosis and to highlight that PN dependence is variable and gene‐specific.^1^, ^2^, ^6^, ^7^, ^8^, ^9^ The PCSK1 gene, localized on chromosome 5q15, encodes the proprotein convertase‐1/3 (PC1/3), a calcium‐dependent serine proteases highly expressed in EECs, where it is essential for processing several prohormones and proneuropeptides (including proopiomelanocortin, proinsulin, pro–glucose‐dependent insulinotropic polypeptide [pro‐GIP] and proglucagon) into their biologically active forms, thus regulating nutrient absorption and gut physiology. The pathophysiological basis of the diarrheal phenotype remains incompletely understood, although impaired processing of intestinal peptides involved in nutrient absorption likely plays a role, even in the presence of normal crypt‐villus morphology.^2^

**Table 1:** Overview of major enteroendocrine‐related congenital diarrheal disorders.

Gene	Function	Diarrheal phenotype	Pancreatic/β‐cell involvement	Other systemic features	PN dependence
PCSK1 *(Enteric dysendocrinosis)* ^1^, ^2^	Prohormone convertase involved in the processing of multiple gut and endocrine peptides	Severe neonatal diet‐induced diarrhea due to impaired enteroendocrine peptide processing	Abnormal glucose homeostasis (↑ proinsulin), not primary β‐cell loss	Multiple endocrinopathies (adrenal insufficiency, hypothyroidism, diabetes insipidus); early‐onset obesity after improvement of malabsorption	Early PN often required; most can be weaned by 1–2 years; long‐term endocrine follow‐up needed
NEUROG3 *(Enteric anendocrinosis)* ^6^	Transcription factor for EEC and pancreatic β‐cell development	Severe neonatal diet‐induced diarrhea due to complete EEC loss	Early insulin‐dependent diabetes due to β‐cell agenesis	—	High PN dependence, usually prolonged
RFX6 *(Mitchell–Riley syndrome)* ^7^	Transcription factor regulating EEC differentiation and β‐cell maturation	Generalized malabsorptive diarrhea	Early insulin‐dependent diabetes due to pancreatic hypoplasia	Intestinal atresia, malrotation, intrinsic and extrinsic biliary duct abnormalities	Variable to high PN dependence; often complicated by surgical resections; poor prognosis
ARX *(X‐linked lissencephaly with ambiguous genitalia)* ^8^	Homeobox transcription factor required for neuronal and EEC subpopulation development	Severe congenital diet‐induced diarrhea due to EEC dysgenesis (reduction of GLP‐1 and CCK‐producing EECs)	Variable, β‐cell dysfunction not consistent	Lissencephaly and severe neurologic abnormalities; seizures; ambiguous genitalia	Variable PN dependence
PERCC1 *(Congenital malabsorptive diarrhea 11)* ^9^	Regulator of EEC differentiation	Generalized malabsorptive diarrhea with reduced EECs	—	—	High PN dependence, usually prolonged

*Note*: The table summarizes the major genetic disorders associated with enteroendocrine‐related congenital diarrhea, detailing their diarrheal phenotype, pancreatic or β‐cell involvement, associated systemic features, and degree of PN dependence. Data are derived from previously published case reports and reviews.^1^, ^2^, ^6^, ^7^, ^8^, ^9^

Abbreviations: CCK, cholecystokinin; EEC, enteroendocrine cell; GLP‐1, glucagon‐like peptide‐1; PN, parenteral nutrition.

**Figure 1:**
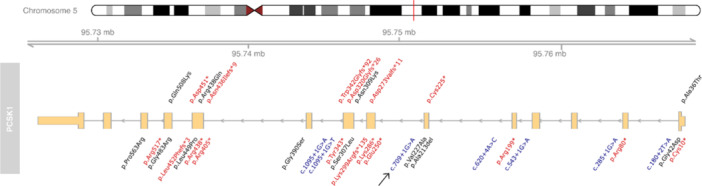
Schematic view of the PCSK1 gene and its known pathogenic variants. Missense or indel variants are reported in black; nonsense and frameshift variants are reported in red and splicing variants are reported in blue. The novel c.709+1 G > A variant is indicated by an arrow. PCSK1, proprotein convertase subtilisin/kexin type 1.

We identified the c.709 + 1 G > A splicing mutation in PCSK1, not previously reported (Figure 1), yet consistent with the established clinical phenotype characterized by early‐onset malabsorptive diarrhea combined with multiple endocrinopathies, including adrenal insufficiency, hypothyroidism, diabetes insipidus, hypogonadism, and early onset obesity—findings that specifically distinguish PCSK1 deficiency from other congenital diarrheal disorders involving enteroendocrine pathways.

Although functional validation by RNA analysis or genetic assays was not feasible, the predicted splicing disruption together with the strong phenotypic concordance provides strong support for the pathogenicity of this variant.

In our case, early genetic analysis was pivotal in minimizing invasive diagnostic procedures such as endoscopy and allowing prompt initiation of nutritional support. Moreover, it enabled proactive endocrine screening, leading to early detection and management of adrenal insufficiency and laying the foundation for ongoing monitoring of additional endocrine complications.

Among disorders affecting EECs, the combination of malabsorptive diarrhea and endocrine abnormalities could suggest autoimmune polyendocrinopathy‐candidiasis‐ectodermal dystrophy (APECED). In APECED, gastrointestinal dysfunction (in ~10% of cases) may precede the classical triad of hypoparathyroidism, adrenal insufficiency, and mucocutaneous candidiasis. Histologically, APECED‐related gastrointestinal involvement is characterized by a marked reduction or absence of EECs;^10^ moreover, the detection of serum anti‐tryptophan hydroxylase (TPH) antibodies supports autoimmune‐mediated EEC destruction,^11^ distinguishing it from PCSK1‐related entericdysendocrinosis, which results from a genetic intrisic dysfunction of EECs.

Currently, no disease‐specific therapy exists for PCSK1 deficiency. Long‐term follow‐up remains crucial to monitor nutritional status, growth, and endocrine function. Although loose stools may persist, most patients are eventually weaned from TPN. However, as malabsorption improves, progressive enteral tolerance often leads to rapid weight gain, typically culminating in early‐onset obesity by age 2–3.^5^

Recently, setmelanotide, a melanocortin‐4 receptor (MC4R) agonist, has been approved for monogenic obesity due to *PCSK1* variants. By mimicking α‐melanocyte‐stimulating hormone (α‐MSH), setmelanotide restores satiety signaling.^12^ However, the early diarrheal phenotype in *PCSK1* deficiency is not mediated by α‐MSH or MC4R pathways but it is thought to result from defective processing of several gut‐derived hormones involved in nutrient absorption, including glucagon‐like peptide‐1 (GLP‐1), GIP, and oxyntomodulin.^2^ Thus, while setmelanotide may represent a promising therapeutic option for later‐onset obesity, its potential impact on neonatal malabsorptive diarrhea remains uncertain, and current management is limited to supportive care.

## 4 CONCLUSION

This case underscores the diagnostic and therapeutic value of early genetic testing in neonates with unexplained severe diarrhea. Prompt identification of PCSK1 deficiency enabled targeted management and endocrine monitoring and informed long‐term follow‐up strategies.

## CONFLICT OF INTEREST STATEMENT

The authors declare no conflicts of interest.

## ETHICS STATEMENT

Parents are aware of the intent to publish and have agreed to it. Written patient consent has been received from the parents and archived. This study did not require Institutional Review Board approval.
